# Tip Discharge Evolution Characteristics and Mechanism Analysis via Optical–Electrical Sensors in Oil-Immersed Transformers

**DOI:** 10.3390/s26010331

**Published:** 2026-01-04

**Authors:** Zehao Chen, Yong Qian, Gehao Sheng, Fenghua Wang, Bing Xue, Chunhui Zhang, Chengxiang Liu

**Affiliations:** 1 School of Electrical Engineering, Shanghai Jiao Tong University, Shanghai 201100, China; chen-z.h@sjtu.edu.cn (Z.C.); shenghe@sjtu.edu.cn (G.S.); fhwang7723@sjtu.edu.cn (F.W.); 2 Hubei Technology Innovation Center for Smart Hydropower, Wuhan 430071, China

**Keywords:** tip discharge, transformer oil, multi-modal sensing, optical–electrical sensors, evolution characteristics, discharge mechanism

## Abstract

Tip discharge in oil-immersed transformers poses a significant threat to insulation integrity. Conventional detection methods, such as gas and electrical analysis, are limited by slow response times or susceptibility to interference. Additionally, the lack of systematic comparisons between aged and fresh oil using multi-modal signal correlations hinders the development of accurate diagnostic strategies. To address this, a multi-modal sensing platform employing optical, UHF, and HFCT sensors, complemented by visual observation, was developed to investigate the evolution characteristics and mechanisms of tip discharge and to compare the detection effectiveness of these methods. Experimental results reveal that aged oil undergoes a novel four-stage evolution, where discharge signals first rise to a local peak, then experience suppression, followed by a dramatic surge, and finally decline slightly before breakdown. This process is governed by an “Impurity-Assisted Cumulative Breakdown Mechanism,” driven by impurity bridge growth and space charge effects, with signal transitions from ‘decoupling’ to synchronization. The optical sensor demonstrated superior sensitivity in early discharge stages compared to electrical methods. In contrast, fresh oil exhibited a “High-Field-Driven Stochastic Breakdown Mechanism,” with isolated pulses from micro-bubble discharges maintaining a metastable state until a critical threshold triggers instantaneous failure. This study enhances the understanding of how oil condition alters discharge mechanisms and underscores the value of multi-modal sensing for insulation condition assessment.

## 1. Introduction

The insulation reliability of oil-immersed transformers is critical for power system stability [1]. Over their service life, local defects inevitably emerge as initiation points for partial discharge (PD), which can progressively lead to insulation failure [2]. Among various PD patterns, tip discharge is particularly representative, typically occurring at sharp metallic edges [3], conductor tips, and geometric irregularities of windings. Its intense localized effects accelerate the degradation of the insulating oil, elevating the risk of breakdown. Therefore, a deep understanding of the evolutionary characteristics and underlying mechanisms of tip discharge is crucial for ensuring the reliable operation of these critical assets.

Monitoring the evolution of tip discharge relies on detecting its associated physical phenomena, such as acoustic, optical, electrical, and chemical signals. Consequently, various conventional sensors are employed, including acoustic sensors, dissolved gas analysis (DGA), ultra-high frequency (UHF) antennas, and high-frequency current transformers (HFCTs). Despite their widespread application, each of these methods suffers from inherent limitations [4,5,6]. For instance, HFCTs target winding discharges but are highly susceptible to ground interference, whereas UHF and acoustic sensors monitor the oil yet are compromised by electromagnetic noise, vibrations, and attenuation. Furthermore, standard DGA methods suffer from excessive response latency. These limitations leave significant detection blind spots. In this context, optical detection offers a unique complementary solution. Due to their compact size, fiber-optic probes can be flexibly deployed throughout the transformer casing or even embedded within windings to assist existing techniques. Crucially, optical sensors possess intrinsic immunity to the diverse spectrum of interferences—electromagnetic, grounding, and mechanical—that plague conventional methods. By serving as a definitive criterion that confirms discharge occurrence (zero false alarms), optical sensing bridges these monitoring gaps. Therefore, an integrated, multi-method approach is essential for achieving robust diagnosis and a comprehensive understanding of discharge evolution.

Optical detection methods have been successfully applied in partial discharge (PD) detection for gas-insulated switchgear (GIS) and are gradually being extended to oil-based discharge studies [7,8,9]. In creeping discharge research, Fan Wenjie et al. revealed the evolution law of PD under oil–paper needle–plate electrodes through multi-technique measurements [10]; Liu Yang et al. established a multi-physical signal platform and proposed a stage division method [11]; and Jia Tao et al. characterized multi-stage features of oil–pressboard interface discharges and their corresponding signals [5]. These studies demonstrate that optical detection has been validated as effective for creeping discharge, with substantial progress in sensitivity, effectiveness, and mechanistic understanding. However, research on tip discharge in oil-immersed transformers remains relatively limited. Some scholars have explored optical detection of tip discharge: Puhan et al. successfully measured optical signals from a needle–plate electrode model in oil, confirming the feasibility of the method [6]; Jia et al. compared pulse current and optical detection, showing that optical signals can serve as a complementary reference to phase-resolved partial discharge (PRPD) patterns from conventional electrical measurements [12]; and Zhu et al. combined HFCT and optical detection for synchronized measurement of tip discharge, analyzing the effect of sensor distance on attenuation and demonstrating higher sensitivity of optical detection at short range [13]. Although these works provide valuable insights into optical detection of tip discharge in oil, they have not yet fully leveraged the dynamic correlations between multi-modal signals to reveal the underlying physical progression. Furthermore, a systematic comparison of the distinct failure mechanisms in aged versus fresh oil is notably absent. Given that operational transformers invariably operate with oil containing impurities, understanding how these conditions fundamentally alter the discharge evolution is crucial for practical diagnostics. Addressing these critical gaps constitutes the core focus of this paper.

The remainder of this paper is organized as follows. Section 2 describes the experimental setup, including the test platform, the needle–plate electrode model, the design and characteristics of the three sensors (optical, UHF, and HFCT), and the experimental procedure. Section 3 presents the experimental results, detailing the evolution of discharge signals in both aged and fresh oil, including phase-resolved pulse waveforms and cumulative statistical data. Section 4 provides a comprehensive analysis of the underlying physical mechanisms, correlating the multi-modal sensing data with visual observations and theoretical force analysis to explain the distinct evolution patterns. Finally, Section 5 concludes the paper by summarizing the main findings and their implications.

## 2. Experimental Setup

### 2.1. Experimental System

**Figure 1:**
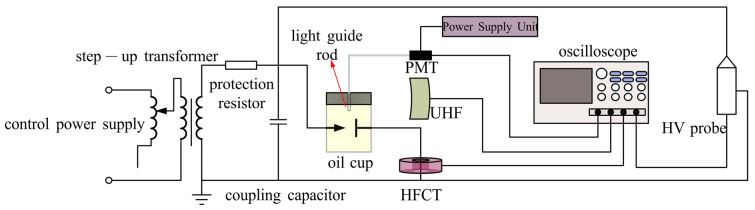
Principle circuit of the tip discharge experimental platform.

Figure 1 illustrates the high-voltage experimental platform established in this study. The system consists of a control power supply, a step-up transformer (maximum output: 150 kV, 50 Hz), a protection resistor (R = 10 kΩ), a coupling capacitor, an oil cup containing the tip-defect model, three types of sensors, a high-voltage probe (Tektronix-P6015A, Shanghai, China), and a digital oscilloscope (RIGOL-DHO5104, Beijing, China). The entire setup complies with the requirements of IEC 60270. The protection resistor is connected between the step-up transformer and the coupling capacitor to ensure circuit safety. The optical radiation, electromagnetic waves, and discharge current generated by partial discharges are simultaneously captured by an optical sensor (quartz light guide rod with a photoelectric converter), a UHF sensor, and an HFCT sensor, respectively. The voltage phase is recorded using the high-voltage probe, and all four channels of data are synchronously acquired by the oscilloscope operating in peak-detection mode.

To suppress environmental electromagnetic interference, all experiments were conducted in a shielded room. For measurement safety, the grounding of the primary equipment and the secondary instrumentation was isolated using an isolation transformer, preventing surge currents during tip-discharge breakdown from damaging the oscilloscope and the power modules of the photoelectric converter. To mitigate air-corona interference, all exposed sharp edges of the setup were sealed with silicone putty to suppress unintended corona under elevated voltage. All measuring devices were interconnected via shielded cables.

### 2.2. Tip Defect and Sensors

**Figure 2:**
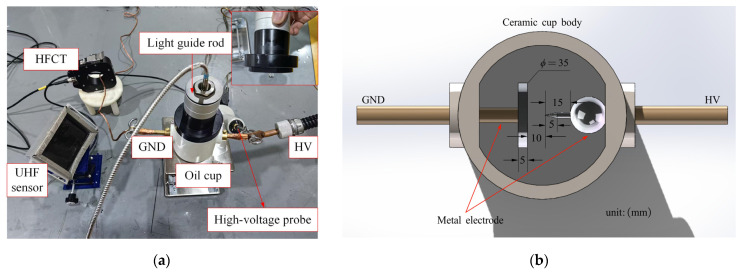
Tip defect and sensors: (**a**) Photograph of the oil cup containing the tip-discharge defect, along with the three types of sensors and the high-voltage probe; (**b**) Needle–plate electrode configuration inside the oil cup simulating the tip discharge, with dimensions labeled according to the actual specimen.

Figure 2a shows the experimental setup. To suppress undesired corona discharges, the sharp edges of the oil cup and the HV probe junction were sealed with silicone putty before testing. A conventional needle-plate electrode configuration was employed inside the oil cup to simulate tip discharge. Figure 2b illustrates the structural model of the oil cup, in which the metallic connecting rod is made of brass, while the electrodes are fabricated from stainless steel. Compared with aluminum or brass electrodes, stainless steel electrodes can withstand longer durations of partial discharge activity and repeated flashover breakdowns. The needle electrode is connected to the high-voltage (HV) terminal, while the plate electrode is grounded (GND). The curvature radius of the needle electrode is approximately 30 μm, the gap distance between the needle and the plate is 10 mm, and the plate electrode has a diameter of 35 mm. All key dimensions are indicated in the figure.

This study employed two types of No. 25 transformer mineral oil to investigate the influence of oil quality on discharge characteristics. The first type was fresh oil, which was purified by a filtration system through degassing and dehydration. It had a moisture content controlled below 15 ppm and was free of measurable solid impurities (0 g). Its breakdown voltage was measured according to the relevant Chinese national standard (utilizing plate-plate electrodes with a 2.5 mm gap and stirring), yielding an average value of 42.7 kV over six tests, which meets the insulation requirements for 110 kV transformers (≥40 kV).

The second type was aged oil, obtained from a 110 kV substation transformer during scheduled replacement after approximately 2.5 to 3 years of operation. Visual inspection confirmed the presence of suspended carbon particles and cellulosic fibers. It is inferred that the carbon particles originated from weak sparks generated during the frequent operation of the on-load tap changer, while the fibers resulted from the mechanical scraping of insulation pressboard or natural shedding due to long-term oil immersion. Quantitative analysis indicated a significant impurity load, with approximately 2–4 g of residue per 500 mL of oil after drying. The moisture content was measured at approximately 35 ppm. Under standard testing conditions, the average breakdown voltage over six tests was 35.2 kV, lower than that of the fresh oil. The partial discharge signals under these two distinct oil conditions are comparatively analyzed in the subsequent experiments.

The primary emission band of tip discharges in mineral oil lies within 300–900 nm [14]. To ensure efficient collection and transmission of light in this range, the optical channel in this study was constructed using a JGS-2 quartz light guide rod in combination with a high-OH quartz optical fiber. The design of the light guide was inspired by our previous work on optical PD detection in GIS [15,16], but in the present study, PMMA was replaced with quartz to meet the requirements of oil-immersed experiments. JGS-2 quartz glass exhibits a transmittance of approximately 93–95% within 300–900 nm and, compared with PMMA, offers superior chemical stability, stronger resistance to corrosion and contamination, making it more suitable for long-term use in oil. The high-OH quartz fiber achieves nearly 99% transmittance in the visible and near-UV ranges with negligible attenuation. Together, they effectively cover the main emission band of tip discharges, providing a reliable basis for optical detection. In terms of placement, the front end of the light guide rod (8 mm in diameter) was aligned perpendicularly to the needle–plate electrode gap, with its center coinciding with the center of the oil cup, thus covering the 10 mm discharge region. The front end of the rod was immersed in oil at a vertical distance of approximately 5 cm from the needle tip to ensure efficient coupling of discharge light into the optical channel.

Once the optical signal enters the photoelectric conversion module, it is converted into an electrical signal, enabling synchronous comparison and analysis with other electrical detection methods. The photoelectric conversion was performed using a HAMAMATSU (Beijing, China) H10722-01 photomultiplier tube (PMT), which has a spectral response range of 230–870 nm and a gain of 10^5^, powered and controlled by the dedicated C10709 module. This configuration covers the main emission band of tip discharges in oil and enables highly sensitive detection of weak optical signals.

The UHF butterfly antenna used in this study was independently designed by our research team. Its basic structure consists of two symmetrical triangular patches connected at a central feed point. As a broadband antenna, the radiation principle of the butterfly antenna is based on the current distribution on the triangular patches, enabling effective reception of UHF signals generated by partial discharges. The antenna substrate is made of polyimide (PI), which provides excellent dielectric properties and mechanical stability, making it suitable for PD detection scenarios. During the design process, CST MICROWAVE STUDIO (2023 version) was employed to perform electromagnetic simulations, determining the antenna’s bandwidth, impedance matching, and radiation performance.

**Figure 3:**
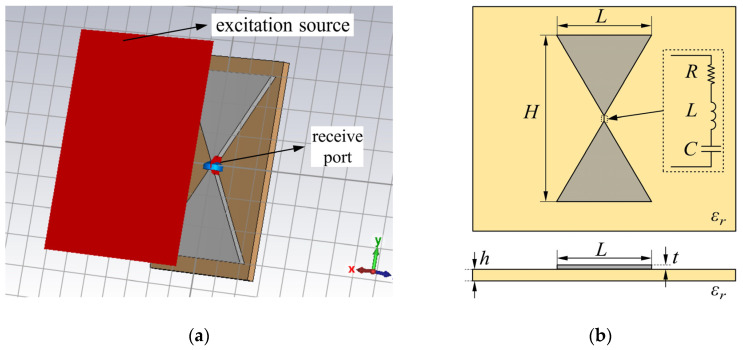
Design and simulation of the UHF butterfly antenna: (**a**) CST simulation model; (**b**) schematic structure of the original antenna.

Figure 3a presents the CST simulation setup, which models the process of incident electromagnetic waves being received by the butterfly antenna and the induced voltage and current being extracted at the feed port. In the simplified schematic, the gray X-shaped structure represents the butterfly antenna patches, consisting of two symmetrical triangular conductors; the brown substrate corresponds to the dielectric base plate (PI material in the actual design); the red block denotes the excitation source, defined in CST as an external wave source illuminating the antenna to test its receiving performance; and the blue block marks the receive port, which is the feed terminal where the induced electrical signal is extracted. Figure 3b illustrates the schematic of the original antenna structure, with key parameters such as patch length, width, apex angle, and feed point position indicated. In practical design, our team iteratively adjusted the slotting of the antenna while running CST simulations, so that the current path length and impedance matching could be optimized to achieve the desired operating frequency band. Since the slotting process involves multiple design steps, only the initial butterfly antenna geometry is presented in the figure for clarity.

**Figure 4:**
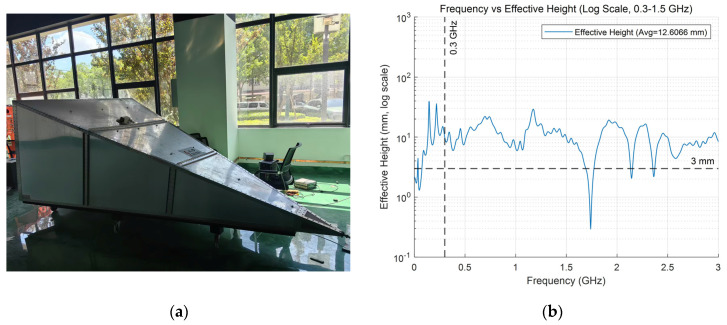
Measurement of antenna performance: (**a**) GTEM cell used for calibration; (**b**) measured effective height of the UHF butterfly antenna.

To evaluate the receiving performance of the designed butterfly antenna, calibration experiments were carried out in a GTEM cell. Figure 4a shows the GTEM cell used in this study, which provides an approximately uniform electric field distribution over a wide frequency range. The antenna was placed near the cell aperture, and by receiving an electric field of known amplitude, the effective height *h*_eff_ was calculated from the measured antenna output voltage *V*:

(1) $$h_{\mathrm{eff}}=\frac{V}{E}$$

where *E* denotes the electric field strength inside the GTEM cell.

Figure 4b presents the measured effective height of the antenna within the frequency range up to 3 GHz. A larger effective height represents stronger coupling to the external field and thus higher sensitivity. According to the Chinese standard for UHF PD sensors, the average effective height in the 300–1500 MHz range should not be less than 8 mm, and the minimum value should not be lower than 3 mm. The measurement results demonstrate that the designed antenna fully satisfies these requirements. Furthermore, as reported in [17], the key frequency range of partial discharges in transformer oil lies below 1 GHz, particularly within 400–900 MHz. In this range, the proposed antenna demonstrates superior and more stable performance compared to higher frequencies, which is consistent with the literature findings and confirms its suitability for tip discharge detection in oil-immersed transformers.

**Figure 5:**
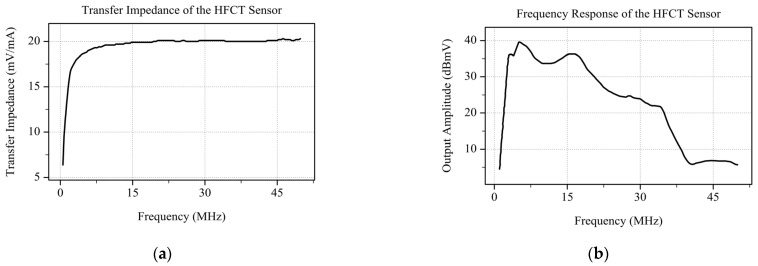
Performance characterization of the HFCT sensor: (**a**) Transfer impedance curve; (**b**) Frequency response and bandwidth.

Figure 5 presents the performance characteristics of the HFCT sensor independently designed and fabricated by our team, including transfer impedance and frequency response. The transfer impedance of the HFCT is defined as

(2) $$Z_{t}(f)=\frac{V_{out}(f)}{I_{in}(f)}$$

where V_out_ (*f*) is the output voltage of the sensor and *I*_in_ (*f*) is the input current passing through it. As shown in Figure 5a, within the frequency range of 3–30 MHz, the transfer impedance remains stable at approximately 20 mV/mA, which is significantly higher than the minimum value of 5 mV/mA required by the Chinese national standard, indicating high sensitivity of the HFCT. Figure 5b shows the frequency response of the HFCT. The results demonstrate that the maximum output corresponds to a center frequency of about 10 MHz, and the effective bandwidth exceeds 2 MHz, meeting the Chinese national standard requirement that “the maximum output frequency should lie within 3–30 MHz, and the bandwidth should not be less than 2 MHz.” These results confirm that the developed HFCT not only meets but also outperforms the relevant technical specifications, while effectively covering the relatively wide spectral range of partial discharge signals, making it suitable for reliable PD current signal detection.

In summary, the optical and electrical sensors selected or custom-designed by our team are all well-suited for detecting tip discharge in transformer oil and demonstrate excellent performance. For the present experiment, each sensor was arranged to achieve its optimal sensing state: the quartz light guide rod was aligned directly with the discharge gap, the UHF antenna was placed in close proximity to the discharge source (given that the ceramic cup exhibits only minor attenuation of UHF signals), and the HFCT was coupled to the sole grounding lead of the oil cup to capture high-frequency current pulses. It should be noted, however, that such ideal arrangements are hardly achievable in practical applications. In actual transformers, the light guide cannot be perfectly aligned with the discharge spot, the UHF antenna cannot be positioned so close to the source, and multiple grounding paths and return circuits exist, which complicate HFCT measurements.

### 2.3. Experimental Procedure

**Figure 6:**
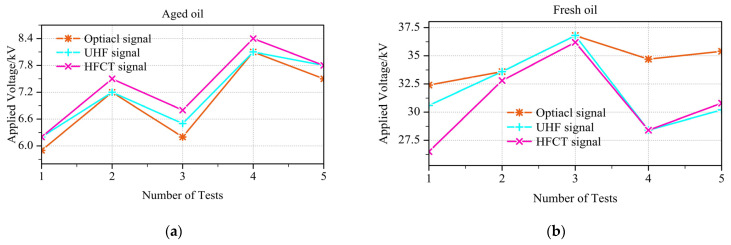
Partial discharge inception voltage (PDIV) measured by optical, UHF, and HFCT methods: (**a**) aged oil; (**b**) fresh oil.

In this study, preliminary experiments were first conducted to measure the partial discharge inception voltage (PDIV) of aged oil and fresh oil using three detection methods. During the tests, the applied voltage was continuously increased at a rate of 1 kV per 30 s until PDIV occurred. The comparative results are shown in Figure 6.

Over five repeated experiments, the PDIV values obtained with the three detection methods in aged oil were concentrated within the range of 5.7–8.4 kV. All three methods exhibited similar fluctuation trends. A clear sensitivity ranking was observed: the optical method consistently yielded the lowest PDIV, followed by the UHF method, with the HFCT method being the least sensitive in this case. In fresh oil, the PDIV values measured by the three methods were generally distributed between 26.3 and 37.5 kV. However, the fluctuations and trends were less synchronized than in aged oil. Among the three methods, the HFCT yielded the lowest PDIV, the UHF method gave intermediate values, and the optical method recorded the highest PDIV.

**Figure 7:**
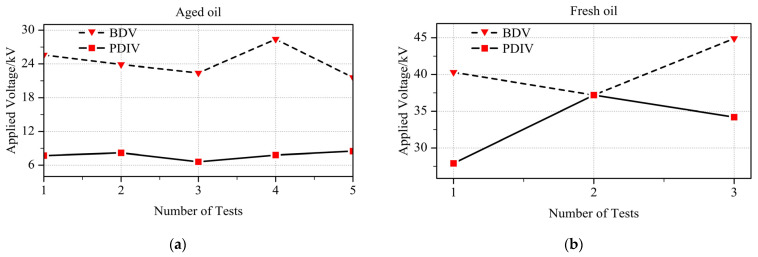
Breakdown voltage (BDV) measured under two oil conditions: (**a**) aged oil, with PDIV determined by the optical method; (**b**) fresh oil, with PDIV determined by the HFCT method.

Before proceeding to the formal experiments, it was necessary to measure the breakdown voltage (BDV) in order to gain a clear reference. Based on the previous PDIV measurements, the most sensitive method was selected for each oil condition: the optical method for aged oil and the HFCT method for fresh oil. Using the same stepwise voltage-rising procedure as described above, five repeated tests were conducted under aged oil and three repeated tests under fresh oil. The results are shown in Figure 7.

From Figure 7, it can be observed that there is no clear correlation between PDIV and BDV. In the case of aged oil, the second test shows PDIV coinciding with BDV, which occurred because the sample underwent sudden breakdown before any distinct inception discharge signal could be detected. For each breakdown test, the oil in the cup was replaced to ensure consistency of the results. Only three tests were conducted with fresh oil, fewer than the five tests with aged oil, because each breakdown in fresh oil produced much larger discharge currents, posing a higher risk of damaging the measuring instruments. Moreover, breakdown in fresh oil generated a considerable amount of carbon black and other byproducts, which made cleaning the oil cup particularly cumbersome.

**Figure 8:**
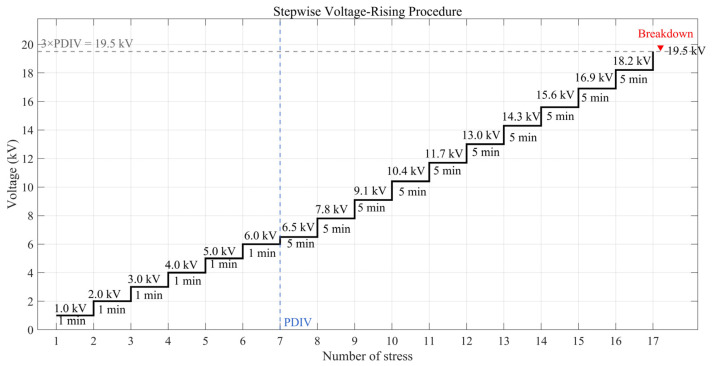
Schematic diagram of the stepwise voltage-rising method in aged oil.

In the subsequent formal experiments, the rising-voltage method specified in IEC 60156 [18] was adopted, as shown in Figure 8, with aged oil as the test medium. At low voltage, the ramp rate was maintained at 1 kV/min until the partial discharge inception voltage (PDIV) of 6.5 kV was reached. Thereafter, the rising step was set to 0.2 times the PDIV every 5 min, with 1 min dwell time before PDIV and 5 min dwell time after PDIV. It is noteworthy that the breakdown did not occur instantaneously at 19.5 kV, but rather during the dwell period at this voltage level, within less than 5 min. During each dwell stage following the PDIV, partial discharge data were synchronously acquired using the three detection methods, together with the phase information recorded by the high-voltage probe. This stepwise rising-voltage method is advantageous, as it not only simulates the typical discharge development process of defect models but also enables the acquisition of a large amount of test data within a relatively short period, thereby meeting the requirements of laboratory investigations.

For fresh oil, the stepwise voltage-rising procedure was consistent with that of aged oil. The PDIV was observed at 27.6 kV, followed by step increments of 0.2 times the PDIV, resulting in voltage levels of 33.1 kV and 38.6 kV. Ultimately, a sudden breakdown occurred when the applied voltage reached 43.7 kV.

## 3. Experimental Results

Corresponding to the stepwise voltage-rising method described above, partial discharge signals were simultaneously recorded by the optical sensor, UHF sensor, and HFCT. For both aged oil and fresh oil, the original discharge data were collected. At each voltage dwell stage, signals corresponding to ten consecutive power-frequency cycles were acquired.

### 3.1. Evolution Characteristics of Partial Discharge in Aged Oil

In the experiments with aged oil, discharge signals were collected over 11 voltage steps. Based on the overall waveform characteristics, these 11 stages are preliminarily categorized into the inception stage, the development stage, and the pre-breakdown stage for description in this section.

#### 3.1.1. Inception Stage in Aged Oil

**Figure 9:**
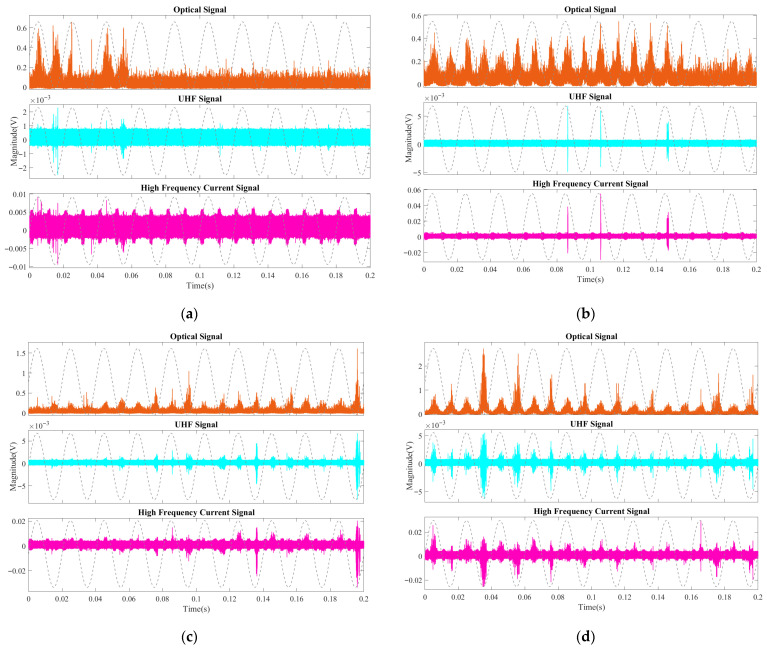
Partial discharge signals in the inception stage with aged oil: (**a**) 1 PDIV (6.5 kV); (**b**) 1.2 PDIV (7.8 kV); (**c**) 1.4 PDIV (9.1 kV); (**d**) 1.6 PDIV (10.4 kV).

As shown in Figure 9a, at 1 PDIV (6.5 kV), the superior sensitivity of the optical method is already evident. The optical sensor captured distinct discharge signals with a high signal-to-noise ratio (SNR), detecting pulses that were either barely discernible or completely missed by the UHF and HFCT sensors. While the optical signal displayed characteristic discharge patterns at 90° and 180° in multiple cycles, the UHF signal showed a distinct discharge only at 180° in specific cycles, and the HFCT signal was heavily obscured by background interference.

This disparity became even more pronounced at 1.2 PDIV (7.8 kV), as shown in Figure 9b. The optical signal revealed multiple clusters of discharge pulses exhibiting typical corona characteristics, which were distributed across 8 power-frequency cycles with a broad phase spread in both positive and negative half-cycles. In sharp contrast, the UHF and HFCT signals were detected in only 3 cycles and appeared as isolated pulses with a significantly narrower phase distribution. This observation confirms that during the inception phase, the optical method possesses a much higher detection probability and sensitivity than conventional electrical methods, effectively capturing early-stage discharge events that produce insufficient electromagnetic radiation or conduction current to be reliably detected.

As the discharge progressed to 1.4 PDIV (9.1 kV) and 1.6 PDIV (10.4 kV) (shown in Figure 9c,d), a marked intensification was observed across all detection channels. Unlike the earlier stages where the optical signal dominated, at these voltage levels, the UHF and HFCT sensors also detected significant and consistent discharge signals.

Crucially, at this stage, the multi-modal signals began to exhibit strong phase correlation. The discharge pulses from the three methods appeared synchronously within the same phase windows, indicating that the discharge had evolved from random, isolated micro-events into a more stable mode along a preferred path. A distinct polarity effect also emerged: the peak-to-peak amplitude of discharge signals in the negative half-cycle was generally higher than that in the positive half-cycle. This trend, characteristic of needle–plate electrode defects where electron emission is facilitated from the negative needle tip, persisted and intensified until the final breakdown.

#### 3.1.2. Development Stage in Aged Oil

**Figure 10:**
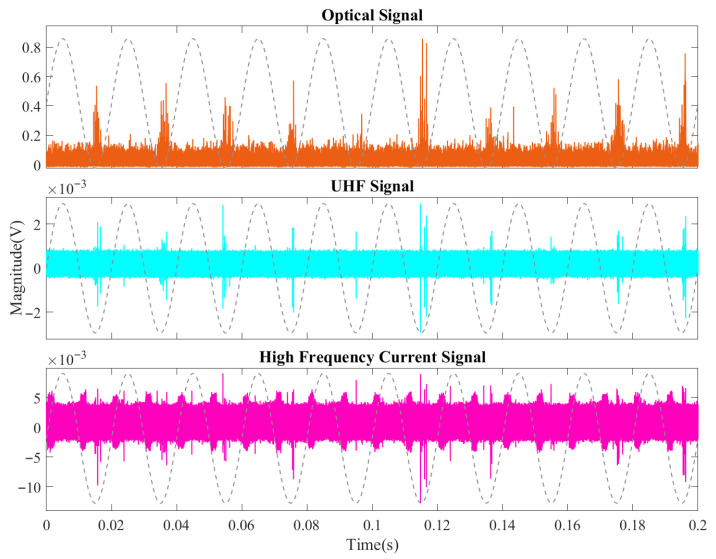
Partial discharge signals in the development stage with aged oil at 2.0 PDIV (13.0 kV).

Figure 10 presents the partial discharge signals at 2.0 PDIV (13.0 kV), which represent the typical characteristics of the suppression stage. It should be noted that the signal patterns at 1.8 PDIV and 2.2 PDIV were found to be highly similar to those at 2.0 PDIV; therefore, only the representative 2.0 PDIV data is presented here. Compared to the previous development stage, a notable reduction in signal intensity is observed. The peak-to-peak values of signals from all three sensors are clearly diminished, and the number of detected discharge pulses has decreased. Most critically, at this voltage level, discharge signals in the positive half-cycle are nearly undetectable across all three methods. This suppression phenomenon, particularly the polarity effect, provides strong evidence for the inhibitory role of space charge accumulation during this phase.

#### 3.1.3. Pre-Breakdown Stage in Aged Oil

**Figure 11:**
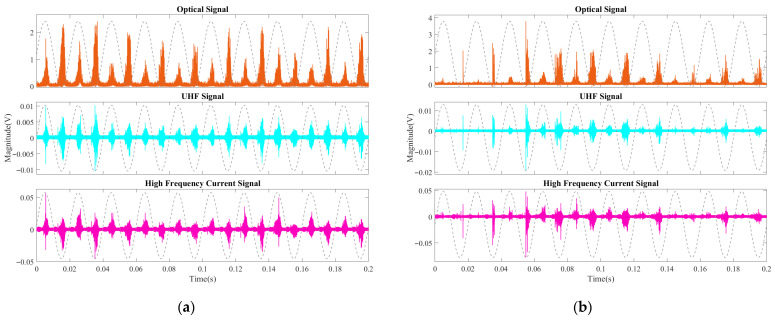
Partial discharge signals in the pre-breakdown stage with aged oil: (**a**) 2.6 PDIV (16.9 kV); (**b**) 3.0 PDIV (19.5 kV).

As the voltage increased further, the discharge entered the pre-breakdown intensification stage. Figure 11a shows the signals at 2.6 PDIV (16.9 kV). The characteristics at 2.4 PDIV were observed to be very similar to those of 2.6 PDIV; therefore, they are not presented here to avoid redundancy. Very distinct clusters of discharge signals with typical corona characteristics were observed, and the signal intensities from all three methods were well-correlated. Notably, the UHF and HFCT signals exhibited high synchronization across all power-frequency cycles, both in terms of intensity and phase, indicating a stable and mature discharge mode.

Figure 11b presents the signals at 3.0 PDIV (19.5 kV), the final stage immediately preceding breakdown. The signals at 2.8 PDIV closely resembled this final state. Breakdown occurred approximately 2–3 min after this data was recorded. In this final transition phase, a critical change was observed: while the peak-to-peak values of some optical and HFCT signals increased, the overall signal count became notably sparser compared to the peak at 2.6 PDIV. This reduction in pulse density serves as a precursor to the formation of a continuous conductive channel just before failure.

### 3.2. Evolution Characteristics of Partial Discharge in Fresh Oil

In the fresh oil experiments, discharge signals were collected over three voltage steps. Unlike the aged oil tests, breakdown occurred after the fourth step, so the discharge stages were not divided as in aged oil.

**Figure 12:**
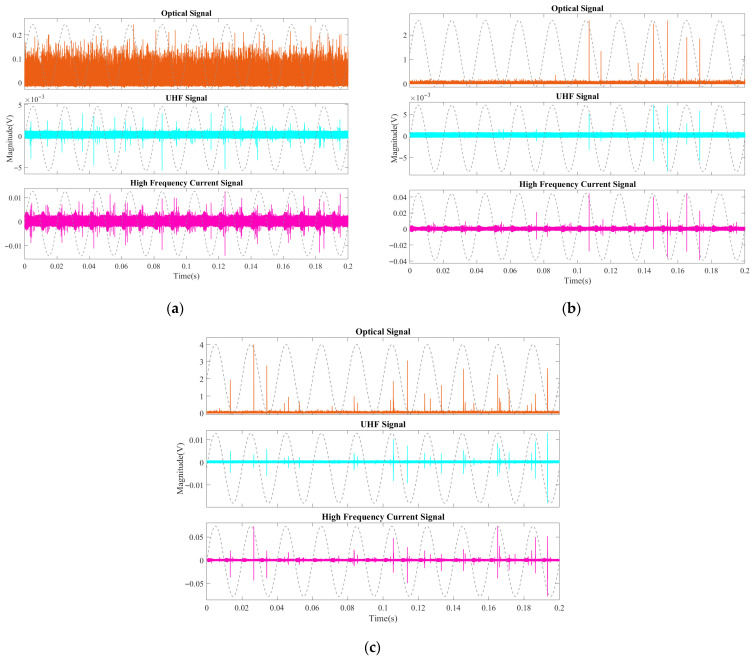
Partial discharge signals in the development stage with fresh oil: (**a**) 1 PDIV (27.6 kV); (**b**) 1.2PDIV (33.1 kV); (**c**) 1.4 PDIV (38.6 kV).

As shown in Figure 12, at 1 PDIV (27.6 kV), only a few weak optical signals were detected, while UHF and HFCT signals were observed, with HFCT showing more signals than UHF. When the voltage increased to 1.2 PDIV (33.1 kV), signals from all three methods appeared, but their numbers were relatively low. Notably, the peak-to-peak values of some optical and HFCT signals were significantly higher than those of the previous stage, while UHF signal peak-to-peak values did not show a marked increase. As the voltage increased to 1.4 PDIV (38.6 kV), the signal behavior followed a similar trend to the previous stage, with a slight increase in signal quantity, though still lower compared to most stages in aged oil. The peak-to-peak values of most signals increased further, with their magnitudes becoming similar to those observed just before breakdown in aged oil. The signals from all three methods were generally consistent in their characteristics.

### 3.3. PD Signal Amplitude and Number in Aged and Fresh Oil

**Figure 13:**
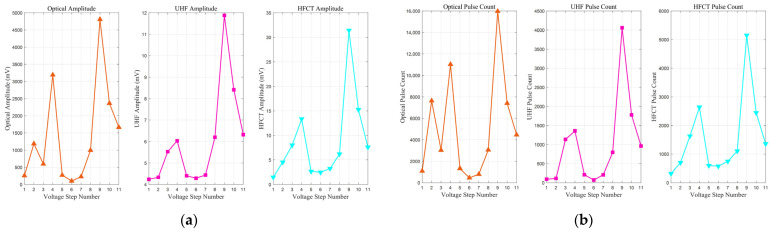
PD signal amplitude and number in Aged Oil: (**a**) amplitude; (**b**) number.

Through synchronous measurements using optical, UHF, and HFCT sensors, it is found that the evolution of tip discharge in aged transformer oil containing impurities follows a distinct non-monotonic pattern under step-wise increasing AC voltage. The entire process can be categorized into four distinct stages: initial development, temporary suppression, pre-breakdown intensification, and final transition. Specifically, the cumulative signal amplitude (Figure 13a) and the total pulse count (Figure 13b) both reach a local maximum at the 4th voltage step, undergo significant suppression between the 5th and 7th steps, surge to a global maximum at the 9th step, and finally decline during the 10th and 11th steps immediately preceding breakdown.

Further analysis from a multi-modal sensing perspective reveals a more profound characteristic of this evolution: a phased transition in signal correlation from ‘decoupling’ in the initial stage to ‘synchronization’ in the mature stage. The distinct physical principles of these sensors are fundamental to interpreting their responses. The optical sensor is sensitive to photon emissions from nearly all ionization and recombination processes, capturing even the most incipient discharge activity. The HFCT measures conducted current pulses, reflecting the magnitude of charge displacement along the discharge path. In contrast, the UHF sensor detects radiated electromagnetic waves, making it selectively sensitive to discharge events with high rates of current change (*di/dt*), which are often indicative of more impulsive discharges. These inherent sensitivities explain the ‘decoupling’ observed during the initial development, which is evident in their divergent dynamic responses. For instance, while both optical and HFCT signals exhibit substantial growth through the first two voltage steps, the UHF signal shows only a marginal increase. More notably, at the third step, the optical signal begins to decline, in direct contrast to the UHF and HFCT signals, which continue to rise. These divergent behaviors are attributed to the presence of various suspended impurities and micro-bubbles within the aged oil, leading to inconsistent sensor responses characteristic of this ‘decoupling’ phase.

Conversely, upon entering the mature evolution stage, the high degree of ‘synchronization’ across all signal trends indicates that the discharge process has transitioned into a unified state dominated by a more macroscopic and deterministic mechanism. At this point, the dominant discharge events produce physical signals that can be stably and synchronously detected by all three types of sensors. Therefore, this characteristic transition from ‘decoupling’ to ‘synchronization’ profoundly elucidates the complete evolutionary process of tip discharge in aged oil: it progresses from a ‘mode-formation’ stage, dominated by local inhomogeneities and characterized by variable physical properties, to a ‘systemic evolution’ stage that can be stably characterized by multi-modal sensing.

**Figure 14:**
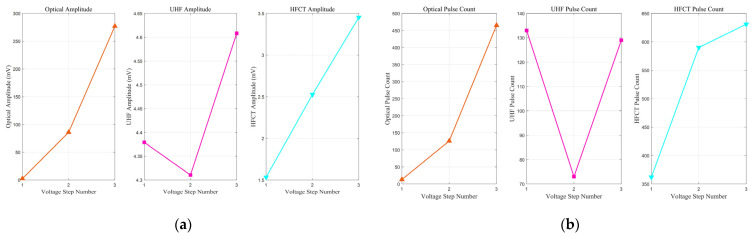
PD signal amplitude and number in fresh oil: (**a**) amplitude; (**b**) number.

In stark contrast to the extended, multi-stage evolution observed in aged oil, the discharge development in fresh oil was significantly abbreviated, culminating in a breakdown during the ramp-up to the fourth voltage step. As depicted in Figure 14, the discharge signals in fresh oil exhibit an even more pronounced ‘decoupling’ characteristic from the outset and do not transition into the subsequent suppression or synchronization phases.

Specifically, from the first to the third voltage step, the optical signals (both amplitude and pulse count) show a rapid, monotonic increase of over two orders of magnitude, indicating a dramatic intensification of luminescent discharge activity. In stark contrast, the UHF signal amplitude remains largely stagnant, and its pulse count paradoxically decreases from the first to the second step. The HFCT signals, meanwhile, show a modest growth intermediate between the other two. This extreme divergence in sensor responses—the explosion of the optical signal versus the quiescence of the UHF signal—is the defining characteristic of the initial discharge phase in fresh oil.

A comparative analysis of the discharge characteristics in fresh versus aged oil leads to two critical conclusions. First, the observation of significant signal ‘decoupling’ in pristine oil provides strong evidence that this phenomenon is an intrinsic feature of the initial stages of tip discharge in oil dielectrics, rather than being solely induced by impurities. Second, the evolutionary paths of the two oil types differ fundamentally. While aged oil undergoes a prolonged, complete four-stage evolution (Initial Development, Suppression, Intensification, and Final Transition), the discharge in fresh oil bypasses these intermediate stages, following a more direct and rapid trajectory toward failure. This suggests that although impurities in aged oil lower the discharge inception voltage, the complex space charge accumulation they facilitate may, to some extent, impede the direct propagation of a breakdown channel, thereby prolonging the evolution process. In contrast, once a discharge channel is initiated in pure oil, its path to breakdown appears more straightforward, leading to a faster and more abrupt failure.

## 4. Discussion

**Figure 15:**
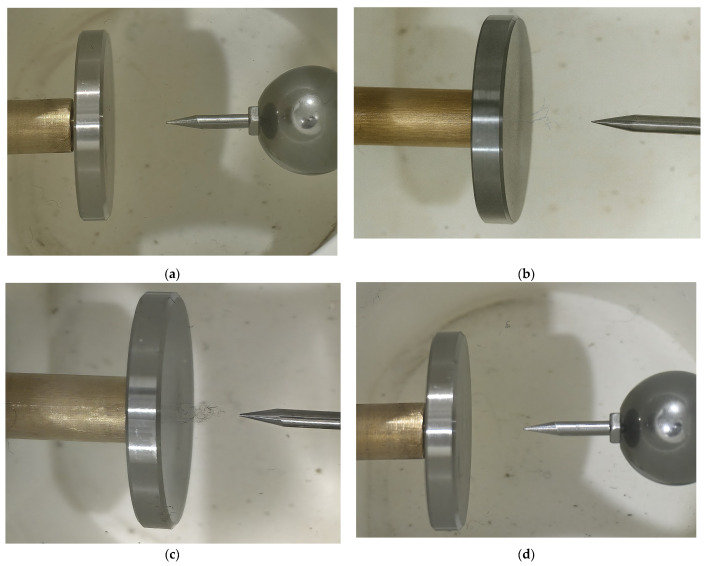
Visual observation of tip discharge process in aged oil at various voltage levels: (**a**) 0 kV; (**b**) 10 kV; (**c**) 15 kV; (**d**) after BDV.

After the experiments in Section 3, the three sensors were removed, and a camera was used to capture part of the tip discharge process in the oil. For this set of visual experiments, the partial discharge inception voltage (PDIV) was 7.3 kV. By combining these images with the discharge signals from Section 3, a more accurate and convincing analysis of the initiation and development of tip discharge in aged oil can be made. The results are shown in Figure 15.

Figure 15a shows a photograph taken before the initiation of the aged oil discharge experiment. It can be observed that several suspended filaments float in the aged oil, with black impurities deposited at the bottom of the oil cup. Despite being aged oil, the oil remains relatively transparent. Figure 15b presents a photo captured at 10 kV, roughly corresponding to voltage steps 2–4 in this experiment. Here, a few suspended filaments are observed in the gap between the needle tip and the center of the plate electrode, aligned parallel to the direction of the electric field, with lengths on the order of a few millimeters. Upon further voltage increase to 18 kV, roughly corresponding to voltage steps 7–9, as shown in Figure 15c, more suspended filaments surround the previously observed filaments, forming a clustered bridge structure. The newly formed filaments range in length from a few hundred micrometers to a few millimeters. As the voltage was increased further, leading to breakdown at 22.4 kV, the appearance of the oil (as shown in Figure 15d) closely resembled that in Figure 15a prior to the application of voltage. After the breakdown, the previously formed filament bridges disappeared, and the oil exhibited no significant degradation or blackening.

In practical applications, transformer oil is inevitably contaminated with various impurities originating from its prolonged interaction with the transformer’s internal components [19]. Numerous studies and extensive field experience have established that fibrous impurities are predominant among these contaminants [20]; for instance, it has been reported that cellulosic particles account for approximately 94% of the total particulate matter in operating transformers [21]. These fibers are primarily shed from the degradation of aging pressboard insulation. Other significant contaminants include: metallic particles, such as copper and iron filings, generated by corrosion or mechanical vibration; carbonaceous particles produced by partial discharges or localized overheating; and gaseous impurities, such as micro-bubbles, formed from the thermal decomposition of the oil-paper system or the vaporization of moisture. The presence and concentration of these impurities are particularly prominent in aged transformers, where long-term operational stresses accelerate material degradation.

While some studies using small particles (e.g., 63–150 μm) suggest that stable cellulosic bridges are difficult to form under AC voltage [19,21], this study demonstrates that the millimeter-scale long fibers naturally present in aged oil can successfully form such structures. Notably, the bridge structure observed in this work (Figure 15b) appears to be discontinuous, particularly near its root, a finding that is highly consistent with the observations in Ref. [22]. This suggests that the pre-existing long fibers, rather than small particles, are the critical factor enabling the formation of these discontinuous impurity bridges under AC voltage.

To physically clarify the motion mechanism of the fibrous impurities, a dynamic analysis of the forces involved is conducted. Based on experimental observations, the millimeter-scale fibers of interest were suspended in the oil prior to voltage application, indicating that gravity and buoyancy were effectively balanced. Therefore, the analysis of their dynamic behavior under electric stress can focus on the dominant electric forces and fluid drag. The equation of motion for a fiber particle can be simplified to [23,24]:

*m*_*p*_*a*_*p*_ = *F*_*DEP*_ + *F*_*C*_ + *F*_*drag*_ (3)

where $m_{p}$ and $a_{p}$ are the mass and acceleration of the fiber particle, respectively. The dominant forces are analyzed as follows:

Dielectrophoretic Force (*F_DEP_*):

(4) $$F_{DEP}=K_{dep}\nabla E^{2}$$

This force is the core driver of the fiber bridge formation. It acts on polarizable, neutral fibers, always directing them towards the region of the strongest electric field gradient (i.e., the needle tip). The coefficient *K_dep_* is a positive value dependent on the fiber geometry (length, diameter) and dielectric properties. For elongated fibers, *K_dep_* is larger, causing them to experience a stronger attractive force.

Viscous Drag (*F_drag_*):

*F*_*drag*_ = −*K*_*d*_*μu* (5)

This force represents the resistance from the transformer oil (with dynamic viscosity *μ*) on a fiber moving at velocity *u*, acting opposite to the direction of motion. The drag coefficient *K_d_* depends on the fiber’s shape and size. This force governs the rate of bridge formation.

Coulomb Force (*F_C_*):

*F_C_* = *qE* (6)

This force acts on impurities carrying a net charge *q* and is considered a secondary effect in the bridging process in this study.

Governed by Equation (4), the dominant dielectrophoretic force (*F_DEP_*) scales with fiber length. Longer fibers experience stronger attraction, overcoming viscous drag (*F_drag_*) and secondary Coulomb effects (*F_C_*) to rapidly establish the initial bridge skeleton. Subsequently, shorter fibers with weaker driving forces migrate more slowly and accumulate on this structure. This mechanism quantitatively explains the observed experimental sequence: the rapid formation of a thin filamentary skeleton followed by gradual bridge thickening.

**Figure 16:**
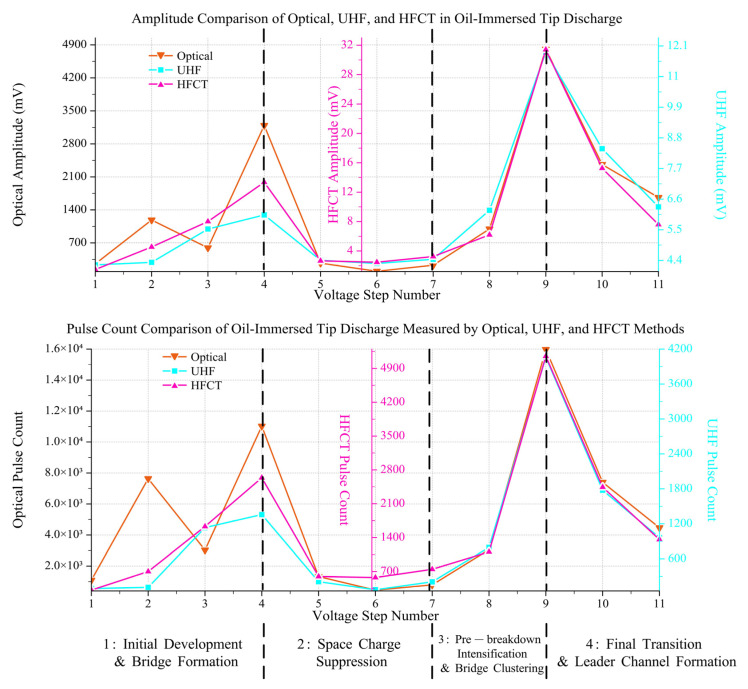
Multi-modal analysis of the evolution of tip discharge characteristics in aged oil using optical–electrical sensors. The cumulative signal amplitude and total pulse count from the optical, UHF, and HFCT sensors are presented on comparable scales to highlight the four distinct evolutionary stages.

We term the discharge mechanism in aged oil as the ‘Impurity-Assisted Cumulative Breakdown Mechanism.’ By correlating the multi-modal sensing data (Figure 13), phase-resolved signals (Figure 9, Figure 10 and Figure 11), and visual observations (Figure 15), we identified that the core of this mechanism lies in the ‘filament bridge’ formed by suspended impurities and the subsequent modulation by the ‘space charge effect.’ These factors collectively govern the observed four-stage non-monotonic evolution of the discharge. The dynamic interplay and evolution of the forces acting on these impurities—analyzed below—correspond perfectly to these four developmental stages, as synthesized in the physical model shown in Figure 16.

1: Initial Development & Bridge Formation (Steps 1–4, 1.0–1.6 PDIV)

This stage is characterized by the formation of the impurity bridge, corresponding to the rapid growth and significant ‘decoupling’ of discharge signals. Physically, the strong dielectrophoretic force (*F_DEP_*) overcomes the oil’s viscous drag (*F_drag_*), capturing and pulling dispersed long fibers into the inter-electrode space to gradually form a discontinuous initial bridge structure (as seen in Figure 15b). Discharges then initiate along these still-unstable fibrous paths in the form of weak coronas. This discharge mode perfectly explains the ‘decoupling’ phenomenon: weak coronas produce sufficient photons, making the optical signal the most prominent, but their low rate of current change (*di/dt*) and small charge magnitude result in weak electromagnetic radiation and conducted currents, leading to a lagging response from the UHF and HFCT sensors.

2: Space Charge Suppression (Steps 4–7, 1.6–2.0 PDIV)

In this stage, all discharge signals decrease synchronously. The physical mechanism is the accumulation of space charge injected from the copious discharges in Stage 1, creating a shielding effect that weakens the local effective electric field *E*. According to the equations, a reduction in the local field *E* directly causes a sharp decrease in the driving force *F_DEP_*, thus inhibiting further bridge development and discharge initiation. At this point, the discharge activity is governed by a single, macroscopic mechanism: the space charge shielding effect. This global effect suppresses the initiation of all discharge modes uniformly, causing the optical, UHF, and HFCT signals to decay in unison and marking the transition into the ‘synchronization’ phase.

3: Pre-breakdown Intensification & Bridge Clustering (Steps 7–9, 2.0–2.6 PDIV)

When the applied voltage becomes high enough to overcome the space charge shielding, the local field *E* intensifies again. Now, *F_DEP_* once again becomes absolutely dominant, attracting more impurities and making the fiber bridge thicker and more stable, forming the clustered structure observed visually (Figure 15c). This stable, low-insulation-strength channel causes the discharge mode to transition from weak coronas to higher-energy, more impulsive streamer discharges. These strong streamers simultaneously produce intense photon emissions, large conducted currents, and high *di/dt*, allowing them to be efficiently detected by all three sensors, which grow explosively and synchronously to the global maximum.

4: Final Transition & Leader Channel Formation (Steps 9–11, 2.6–3.0 PDIV)

The signal decay observed in the fourth stage, following the pre-breakdown peak, signifies a fundamental transition in the discharge mode. In Stage 3, the intense signals originate from high-frequency, pulsatile discharges within the tiny oil gap between the tip of the formed impurity bridge and the needle electrode. As the process enters Stage 4, this final gap is bridged, establishing a direct connection between the impurity structure and the needle tip. This connection creates a relatively stable, low-impedance path spanning the electrode gap. Consequently, the nature of the current flow shifts from discrete, high-*di/dt* pulses to a more continuous, quasi-DC leakage current, forming an ‘incipient leader channel’. Because PD detection systems are inherently insensitive to such quasi-continuous currents, the measured pulse count and amplitude decrease significantly. Therefore, this signal decay does not signify a reduction in discharge activity but rather indicates the formation of a decisive, conductive leader channel, serving as an immediate precursor to complete breakdown.

**Figure 17:**
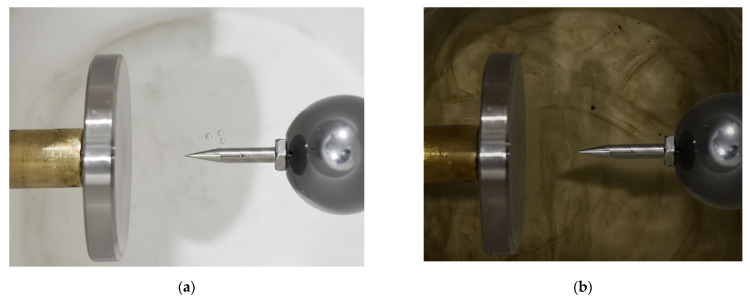
Visual observation of tip discharge process in fresh oil: (**a**) 32.5 kV; (**b**) after BDV.

To validate the failure mechanism in fresh oil, a separate visual observation of its discharge process was conducted. In this specific experiment, the partial discharge inception voltage (PDIV) was recorded at 27.1 kV, and the final breakdown voltage (BDV) occurred at 46.2 kV. The visual observations, presented in Figure 17, provide compelling evidence that contradicts the progressive evolution model seen in aged oil, pointing instead to a stochastic, bubble-initiated breakdown mechanism.

Figure 17a captures the state at 32.5 kV (approximately 1.2 PDIV). Despite the voltage being well above inception, the electrode gap remains visually clear, with no formation of stable structures such as the impurity bridges observed in aged oil. Careful observation revealed only sporadic micro-bubbles rising rapidly from the needle tip due to buoyancy. This lack of macroscopic evolution confirms that the pre-breakdown phase in fresh oil does not involve the gradual buildup of a conductive path. Instead, the discharge remains in a metastable state driven by the random generation of transient micro-bubbles in the high-field region.

Figure 17b shows the aftermath immediately following the breakdown. The oil instantaneously turned black, filled with carbonaceous particles generated by intense pyrolysis. Crucially, no massive accumulation of bubbles was observed post-breakdown. This phenomenon indicates that the failure was not a result of a sustained, gas-generating partial discharge process (which would produce a bubble cloud), but rather a “hard,” instantaneous streamer breakdown. Once a critical micro-bubble condition was met, a high-velocity streamer traversed the gap in nanoseconds, releasing massive energy and causing instant carbonization without a prolonged precursor phase. Thus, the visual evidence confirms that fresh oil exhibits an abbreviated failure mode: a stochastic inception followed directly by catastrophic breakdown, bypassing the intermediate suppression or intensification stages characteristic of aged oil.

We term the discharge mechanism in fresh oil as the “High-Field-Driven Stochastic Breakdown Mechanism”. To elucidate the physical mechanism underlying these observations, a comprehensive analysis of the tip discharge process in fresh oil is presented below.

1: Bubble Formation and Internal Micro-Discharges (Governed by Bubble Theory, Steps 1~before BDV)

**Figure 18:**
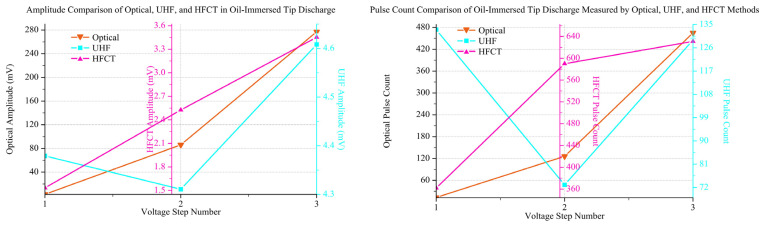
Multi-modal analysis of the evolution of tip discharge characteristics in fresh oil using optical–electrical sensors. The cumulative signal amplitude and pulse count are shown on comparable scales.

As shown in Figure 17 and Figure 18, the entire recordable evolution, spanning from the first to the third voltage step, falls completely within this initial stage. This stage corresponds to the pronounced ‘decoupling’ of the sensor signals. The direct observation of “small bubbles” confirms that the discharge is initiated by bubble formation. As theory suggests, the high electric field at the needle tip causes localized oil vaporization or decomposition, creating bubbles with a much lower permittivity than the oil. The electric field is intensely enhanced inside these bubbles, causing discharges to occur preferentially within them.

This explains the extreme divergence in the sensor signals: on one hand, a massive number of visually imperceptible micro-bubbles may have been generated, and the weak discharges within them were captured by the highly sensitive optical sensor, leading to its explosive signal growth. On the other hand, the energy of a discharge even within the few macroscopically visible bubbles that were occasionally observed is extremely low (low *di/dt*), resulting in inefficient electromagnetic radiation, which explains the quiescent response of the UHF sensor. The apparent contradiction between “no significant visual change” and the dramatic increase in optical signals indicates that the dominant process is occurring at the microscopic scale.

2: Transient Channel Formation and Streamer Breakdown (Governed by Electron Collision Ionization Theory)

The second stage is the instantaneous process of final breakdown, which occurs once the voltage reaches a critical threshold and therefore cannot be captured by the step-wise data acquisition system. We infer that its physical mechanism differs fundamentally from the slow coalescence of visible, drifting bubbles. Instead, based on the streamer theory and the observed instantaneous carbonization, the process is likely driven by the rapid generation of micro-bubbles in the high-stress region. At the critical field intensity, these micro-bubbles are presumed to form a transient, continuous, low-density gaseous channel across the electrode gap in a matter of nanoseconds. While this process is too rapid and microscopic to be visually perceived, it creates a favorable path for high-velocity streamer propagation.

Once this transient channel is established, electrons are rapidly accelerated within it, gaining sufficient energy to cause an electron avalanche via collisions. This quickly develops into a streamer that traverses the gap, causing instantaneous insulation failure. The final, high-energy plasma channel of the breakdown arc intensely pyrolyzes the surrounding oil molecules. This mechanism offers a consistent physical explanation for the observation that the oil “instantaneously turned black, producing a large number of carbonaceous particles” without the prior formation of a stable bubble bridge.

The principal findings of this study, contrasting the evolution characteristics and physical mechanisms for both oil types, are summarized in Table 1.

## 5. Conclusions

Based on a custom-designed multi-modal optical–electrical sensing platform, this paper systematically investigates the evolution characteristics and mechanism of tip discharge in aged and fresh transformer oil under AC voltage. By synchronously acquiring optical, UHF, and HFCT signals, the study captures the complete discharge process from inception to breakdown, establishing distinct evolutionary models for both oil conditions supported by visual observations and theoretical analysis.

In aged oil, a non-monotonic four-stage evolution characteristic was identified: (1) Initial Development & Bridge Formation, (2) Space Charge Suppression, (3) Pre-breakdown Intensification & Bridge Clustering, and (4) Final Transition & Leader Channel Formation. Mechanistically, this sustained process is defined as the “Impurity-Assisted Cumulative Breakdown Mechanism,” which is driven by the dielectrophoretic force acting on impurity fibers and modulated by space charge shielding. From a sensing perspective, the optical method proved most sensitive during the initial stage, capturing weak corona discharges along the forming bridge that were undetectable by electrical sensors (the ‘decoupling’ phase). As the bridge stabilized and space charge exerted a unified suppression effect across the gap, the multi-modal signals transitioned to a ‘synchronized’ state, indicating the defect had evolved from randomized micro-events to a deterministic process.

In contrast, the process in fresh oil is defined as the “High-Field-Driven Stochastic Breakdown Mechanism,” where the entire recordable evolution is characterized by fewer, relatively isolated, high-amplitude pulses from micro-bubble discharges, differing significantly from the clustered pulse bursts observed in aged oil. The final breakdown is driven by the rapid formation of a transient micro-bubble channel and high-speed streamer. Throughout the recordable phase, persistent signal ‘decoupling’ was observed. While the optical method showed lower initial sensitivity, the UHF response fluctuated non-monotonically and failed to track the discharge intensification, rendering it less effective for severity assessment than the Optical and HFCT methods. This comparison reveals that impurities fundamentally alter the discharge pathway from a stochastic process of isolated, bubble-initiated discharges to a prolonged, quasi-stable evolution governed by a physical bridge.

This study underscores the engineering value of multi-modal sensing, particularly the role of optical detection in providing interference-free, reliable monitoring across the entire discharge evolution. Future work will focus on deploying compact optical probes within transformer windings for precise localization and developing fusion algorithms to recognize complex defect types.

## Figures and Tables

**Table 1:** Comparative Summary of Discharge Evolution and Mechanisms in Aged and Fresh Oil.

Characteristic	Aged Oil (Containing Impurities)	Fresh Oil
Evolutionary Pattern	A complete, four-stage process: Initial Development & Bridge Formation; Space Charge Suppression; Pre-breakdown Intensification & Bridge Clustering; Final Transition & Leader Channel Formation.	An abbreviated process comprising: A recordable stage of Bubble Formation and Internal Micro-Discharges; An instantaneous stage of Transient Channel Formation and Streamer Breakdown.
Dominant Physical Mechanism	Formation of a solid filamentary bridge from impurities, governed by Dielectrophoretic (DEP) force and modulated by space charge effects.	Stochastic micro-bubble generation triggering a transient gaseous channel for high-speed streamer propagation.
Key Visual Phenomena	Progressive formation of a visible, clustered filament bridge that disappears after breakdown. No significant oil degradation.	Sporadic visible bubbles with no other macroscopic changes, followed by instantaneous oil blackening (carbonaceous particles) upon breakdown.
Multi-modal Signal Characteristics	Clear transition from signal ‘decoupling’ (Stage 1) to signal ‘synchronization’ (Stages 2–4).	Pronounced and persistent signal ‘decoupling’ throughout the entire recordable phase.
Final Transition to Breakdown	Formation of a conductive leader channel along the completed bridge, indicated by a decrease in PD signal count before breakdown.	Formation of a transient micro-bubble channel enabling a high-speed streamer breakdown without a recordable precursor phase.

## Data Availability

Data will be made available from the corresponding author upon reasonable request.
